# Sclerosing Stromal Tumor: A Rare Ovarian Neoplasm

**Published:** 2017-10-01

**Authors:** Shilpa Bairwa, Rahul Narayan Satarkar, Shivani Kalhan, Shilpa Garg, Ashok Sangwaiya, Pawan Singh

**Affiliations:** *Dept of Pathology, SHKM, GMC, Nalhar, Mewat, Haryana, India*

**Keywords:** Benign ovarian neoplasm, Pseudolobular pattern, Sclerosing stromal tumor

## Abstract

Ovarian sex cord-stromal tumors are relatively infrequent neoplasms that account for approximately 8% of all primary ovarian neoplasm. Sex cord-stromal tumors of the ovary include granulosa cell tumors, fibrothecomas, Sertoli-Leydigcell tumors, steroid cell tumors, and sclerosing stromal tumors (SST). Sclerosing stromal tumors account for 2% to 6% of ovarian stromal tumors. Despite the rarity of this particular neoplasm, it is not always possible to predict the presence of this tumor preoperatively on the basis of clinical and sonographic findings. Histopathological and immunohistochemical (IHC) examinations confirm the diagnosis. Herein, the clinical findings and histopathological features of SST are described in a 24-year-old female.

## Introduction

Sclerosing stromal tumor (SST) is an extremely rare benign ovarian neoplasm. It is a subtype of ovarian stromal neoplasm of sex chord-stromal origin that has distinctive clinical and pathological features, which differ from those of other stromal tumors (1). SST accounts for 2% to 6% of ovarian stromal tumors. These tumors occur predominantly in the second and third decade of life(2). Patients usually present with menstrual irregularities, pelvic pain, and an abdominal mass. SSTs are mostly hormonally inactive. If hormonally active, they are usually androgenic and occur most frequently during pregnancy(3). Fewer than 208 cases withSSTs are described in the literature since its first description in 1973 by Chalvardijan and Scully, indicating the rarity of this entity(4). 

Histopathological and immunohistochemical (IHC) examinations confirm the diagnosis. Herein, the clinical findings and histopathological features of SST are described in a 24-year-old female.

## Casereport

 A 24-year-old mother presented with menstrual irregularities and dull aching lower abdominal pain. On physical examination, a firm mass in the hypogastric region was noted. Pelvic ultrasonography showed a right adnexal, solid cystic mass measuring 9.5x8.5 cm with normal left ovary, uterus and endometrium. All laboratory investigations including tumor markers and serum hormonal assays were within normal limits. Unilateral salpingo-oophorectomywas conductedand biopsies from the omentum, peritoneum, and left ovary were taken.

**Figure 1:**
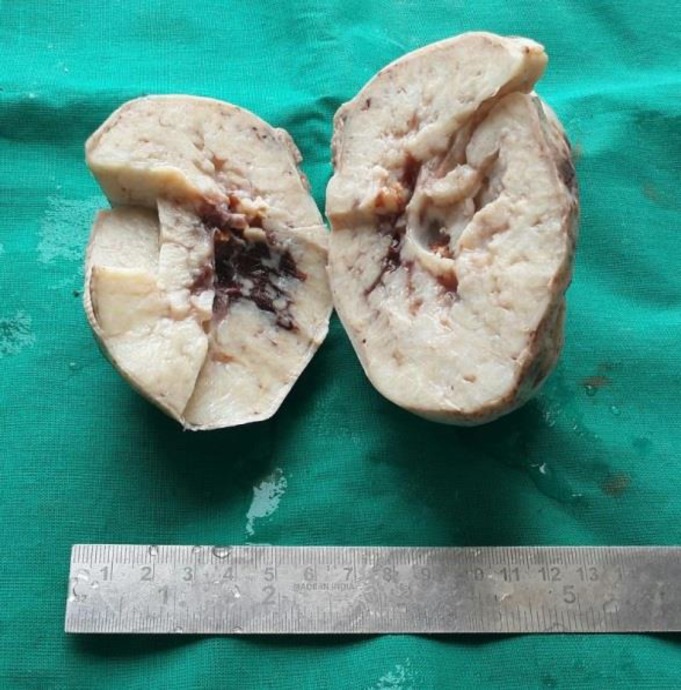
Photograph ofencapsulated, globular mass with grey white to yellowish, solid cut surface

The gross examination of the resected specimen showed an encapsulated, globular mass measuring 10x10x8 cm with an elongated fallopian tube measuring 7.5 cm in length. The external surface was smooth and intact. Cut surface was grey white to yellowish, solid with a rubbery consistency and small cystic spaces (Figure 1).

 No hemorrhage or necrosis was noted. 

**Figure 2:**
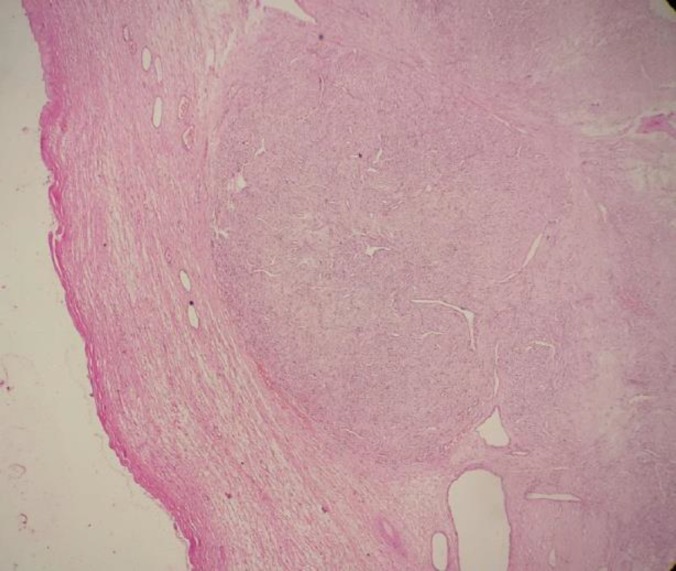
Photomicrograph of a well-encapsulated tumor witha pseudo-lobular pattern (H&E staining, 100X)

Light microscopic examination revealed a well-encapsulated tumor witha pseudo-lobular pattern (Figure 2).

**Figure 3:**
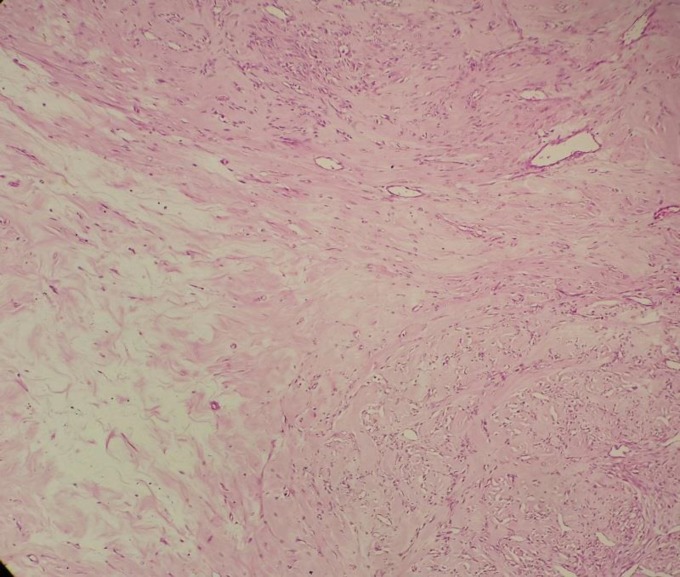
Photomicrograph ofcellular areas separated by edematous, collagenous, and hypocellular areas(H&E staining, 200X)

The cellular areas were separated by edematous, collagenous, and hypocellular areas (Figure 3). 

**Figure 4:**
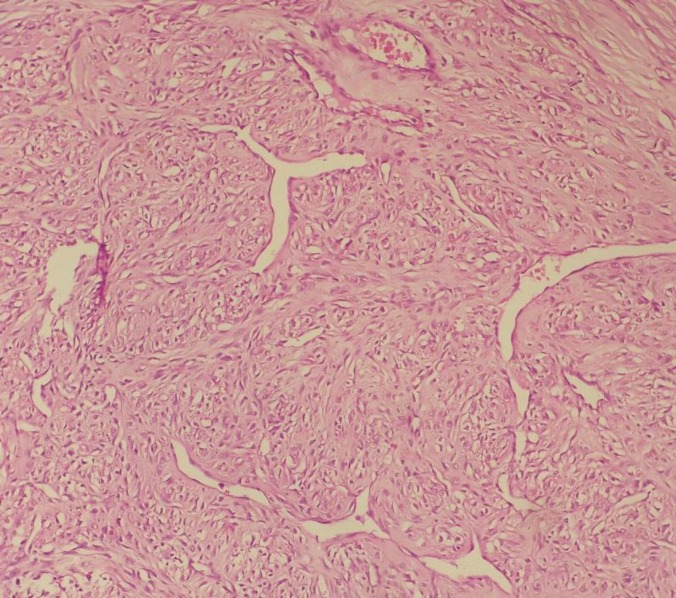
Photomicrograph oftwo cell types, spindle and polygonal, with hemangiopericytomablood vessels(H&E staining, 400X

There were two cell types within the lobules; spindle cells producing collagen and polygonal cells with round to oval nuclei, fine chromatin and prominent nucleoli. Many thin-walled blood vessels, some with branching, simulating a hemangiopericytomatous pattern were also observedin the cellular areas (Figure 4).

 Normal ovarian stroma was not identified in any sections. No mitotic activity was noted. 

Based on the above clinical and histopathological findings, a diagnosis of SST was established.

## Discussion

 Ovarian sex cord-stromal tumors are relatively infrequent neoplasms that account for approximately 8% of all primary ovarian neoplasms(5). Sex cord-stromal tumors of the ovary include granulosa cell tumors, fibrothecomas, Sertoli-Leydig cell tumors, steroid cell tumors, and SSTs (6). SSTs account for only 6% of sex cord-stromal tumors (2). 

The most common clinical symptoms include menstrual irregularities, pelvic pain, and non-specific symptoms related to an ovarian mass(1). Masculinization or anovulation may be present in some patients, as they are occasionally associated with estrogen and androgen secretion(3). SSTs usually present in the 2nd and 3rd decades of life; whereas, other ovarian stromal tumors present in the 5th or 6th decade of life(1).

The etiology of SSTs is not well understood. Ismail et al., proposed the hypothesis that an endocrine milieu might be responsible for the morphology of SSTs and that they may develop from pre-existing ovarian fibromas(7). Damajanov et al., on the basis of ultrastructural features, postulated that SSTs are derived from the pluripotent immature stromal cells of the ovarian cortex(8). However, it is proposed that SSTs are derived from a population of muscle specific actin positive elements from the theca externa, namely, the perifollicular myoid stromal cells. Tiltman and Haffeyee suggested that SSTs and thecoma are probably closely related entities as they share some morphological features and antigenic determinants such assmooth muscle actin and vimentin(9). 

Histologically, it is characterized by a pseudo-lobular arrangement with cellular areas and hypocellular interlobular areas. Cellular areas consist of a dual population of cells comprising of spindled fibroblastic cells and round to oval to polygonal lipid containing cells(4). In some cases, clear cells show signet ring cell morphology. Such cases require differentiation from the Krukenberg tumor. However, they can be differentiated as the latter are mostly bilateral and usually occur in the 6thand 7thdecades of life and lack a pseudo-lobular pattern. The Krukenberg tumor also exhibits mitotic activity and nuclear atypia(2). Cellular areas also show many thin-walled blood vessels mimicking vascular tumors, but inhibin positivity suggests the diagnosis of SSTs(10).

The other main differential diagnoses of SSTs include other sex cord-stromal tumors such as fibroma and thecoma. Sex cord-stromal tumors can be differentiated from SSTs, based on different histopathological findings(4).

Massive ovarian edema might be confused with SSTs. However, this confusion can be resolved by finding the entrapped ovarian tissue within the edematous stroma in massive ovarian edema(11).

On IHC, SSTs show positivity for vimentin, smooth muscle actin, alpha inhibin, calretinin, estrogen receptor (ER) ER, progesterone receptor (PR) PR, and vascular endothelial growth factor (VEGF). They are negative for S100 and epithelial markers(9).

SSTs can be treated successfully by enucleation or unilateral salpingo-oophorectomy. No local or distant recurrences are reported in the literature(12).

## Conclusion

Due to the rarity of SSTs, it is not always possible to predict the presence of this tumor preoperatively on the basis of clinical and sonographic findings. SSTs should be considered in young females with unilateral, solid cystic, complex ovarian mass and related symptoms. It has a benign course and entails a very good prognosis with conservative surgical treatment. Characteristic histopathological features and IHC establish the diagnosis.
